# A Rational Adoption of the High Sensitive Assay for Cardiac Troponin I in Diagnostic Routine

**DOI:** 10.1155/2017/4523096

**Published:** 2017-05-15

**Authors:** Antonio Croce, Pietro Brunati, Carlo Colzani, Riccardo Terramocci, Stefano Favero, Gabriele Bordoni, Claudio Galli

**Affiliations:** ^1^Department of Diagnostic Services, ASST Valtellina e Alto Lario, Sondrio, Italy; ^2^Central Laboratory, Hospital “Valduce”, Como, Italy; ^3^Medical Scientific Liaison Europe, Abbott Diagnostics, Roma, Italy

## Abstract

We describe the adoption of high sensitive troponin I (hsTnI) in clinical practice in two hospital settings in Italy. Samples from 426 consecutive patients (mean age 68.8 ± 17.0) admitted to the Emergency Department with a suspected acute coronary syndrome (ACS) have been tested at admittance and after 3 and 6 hours by contemporary TnI and hsTnI. Results have been compared to the final clinical diagnosis. Troponin was detectable in 68.6% by TnI and 89.9% by hsTnI. Since hsTnI has a lower threshold for females, 38/41 patients with positive values only by hsTnI were women. The correlation between the assays was very high (*r* = 0.92). A diagnosis of acute myocardial infarction (AMI) was made in 45 cases (10.5%). The negative and positive predictive values for a 50% troponin variation at 3 hours were 95.8% and 66.7% for hsTnI and 95.0% and 52.6% for TnI and at 6 hours 90.3% and 100% for hsTnI and 88.9% and 78.9% for TnI, respectively. Receiver operating characteristic (ROC) curve analysis demonstrated a greater efficiency by hsTnI at 3 hours versus 6 hours (AUC = 0.91 versus 0.72). The main benefits of hsTnI are the adoption of gender-specific 99th percentile and the shortening of time to decision.

## 1. Introduction

Acute myocardial infarction (AMI) is one of the leading causes of death and disability worldwide. Patients with a suspected IMA account for up to 10% of all admittance to the emergency room (ER), though only in 10 to 20% of these a final diagnosis of AMI is posed. A rapid diagnosis, either for ruling in and giving appropriate and timely care or for ruling out, is then necessary [1]. Testing for cardiac troponin (cTn) is of central relevance on both purposes [2, 3], and the adoption of high sensitive assays (hscTn) has been envisioned to speed up the ER process for suspected AMI by reducing the time between the initial and the second draw to 3 hours or even to 1 hour [3].

High sensitive assays for cardiac troponin must fulfill the criteria required by the International Federation for Clinical Chemistry (IFCC) of 10% total imprecision at the 99th percentile of a reference normal population and detection of cTn in at least 50% of individuals belonging to that population [4]. To date, the only commercial assay that fulfills both criteria is the Architect hsTnI (Abbott Laboratories, Wiesbaden, Germany). However, no matter how the assay may be good from an analytical standpoint, its adoption needs quite an adjustment in routine practice, due to several reasons: (a) change of reporting units: hsTnI results shall be reported in ng/L compared to ng/mL [5], (b) time to a second blood draw may be set to 3 hours or even to 1 hour, compared to 6 hours with contemporary methods, and (c) lower thresholds for ruling out and gender-specific thresholds for ruling in.

In view of all this, taking hsTnI in routine practice requires a joint effort by all healthcare professionals acting in the clinical area of AMI. We report here our experience that eventually led to a smooth transition to a new assay as well as to a new diagnostic protocol.

## 2. Patients and Methods

The study has been carried out in three separate phases:

1. Meetings with the chief medical officers and the ED and Cardiology staff at both institutions involved to illustrate the main features of the hsTnI assay compared to the then in use contemporary assay for TnI (Table 1) and to agree on the study protocol. This required blood draws for TnI at admission (T0) and after 3 and 6 hours (T3 and T6), while the protocol in use required only the latter. An additional sampling after 12–24 hours, if deemed necessary, was also included.
2. Upon approval by the local Ethics Committees, a two-month study on all patients admitted to the ED with a suspected acute coronary syndrome (ACS) and who did sign an informed written consent in order to be able to enroll at least 400 cases according to the historical trends has been started.
3. After data reduction and analysis, a final meeting with the aforementioned clinical staff to discuss results and achieve an agreement on the new protocol for ACS diagnosis based on the adoption of hsTnI.

The study protocol envisioned the analysis of fresh lithium heparin plasma samples collected at all the time points by both contemporary and high sensitive assays for cardiac troponin (TnI and hsTnI) on the Abbott ARCHITECT analyzer ci8200 (Como and Sondrio) and ci16200 (Sondrio) (Abbott Diagnostics, Wiesbaden, Germany). The main features of those assays are described in Table 1. Of note, the TnI assay was employed in both hospitals with a diagnostic threshold at 44 ng/L, corresponding to the TnI concentration that in our experience allows attaining a 10% total coefficient of variation (CV), as suggested [5, 6], while for the hsTnI, we adopted gender-specific thresholds according to the manufacturer's indications (16 ng/L for females, 34 ng/L for males; package insert, Abbott ARCHITECT STAT High Sensitive Troponin I). The protocol required all patients with suspected ACS allowed to the ED of the two hospitals during the study period to be tested for cardiac troponin I by TnI and hsTnI at admission (T0) and after 6 hours (T6) that represented the standard of care and additionally after 3 hours after the first draw (T3). Though all results of TnI and hsTnI testing were made available to clinicians, the protocol required that the final diagnosis, as well as clinical decisions, should have been taken according to the standard of care, that is, not taking into account the results for cardiac troponin at T3.

The final diagnosis was made by clinicians in the ED for discharged patients, or by cardiologists or other clinical specialists for the patients admitted to the hospital wards. The analytical evaluation included the imprecision profile of both assays based on the results of internal quality controls (IQC) and the concordance and correlation (linear regression and the Bland–Altman difference plot). The sensitivity, specificity, and overall accuracy of TnI and hsTnI were evaluated according to the relative change (percentage) of troponin levels over time compared to the final diagnosis. For this, we considered as significant a change of 50% or higher, as recently suggested by an Italian consensus paper on the utilization of cardiac troponin for patients with a suspected myocardial infarction without elevation of the ST tract at the electrocardiogram (NSTEMI) [6]. The overall accuracy of both TnI and hsTnI by testing a second sample after 3 or 6 hours has been assessed by receiver operating characteristics (ROC) curves. Finally, survival rates after 18 months from admission have been evaluated by the Kaplan–Meier nonparametric statistics for the 202 patients enrolled at one of the two sites (Sondrio).

Statistical evaluations have been carried out by Analyse-it plug in software (Analyse Ltd, Birmingham, UK) on Excel worksheets and, for the Kaplan–Meier survival curves, by SPSS v23.0 (IBM Italia, Segrate, Italy).

## 3. Results and Discussion

### 3.1. Study Population

During the study period, a total of 8117 patients have been admitted to the ED at the two sites. Out of them, a clinical suspicion of ACS was posed in 426 cases (5.25%) and those were then enrolled in the study.  Table 2 reports the main demographic characteristics of the study population: the data were almost identical at the two sites and are therefore presented together. Cardiovascular risk factors and a history of cardiovascular disease were present in 184 cases (43.2%) and in 115 cases (27.0%), respectively, with no significant differences by gender.

### 3.2. Analytical Results

**Figure 1:**
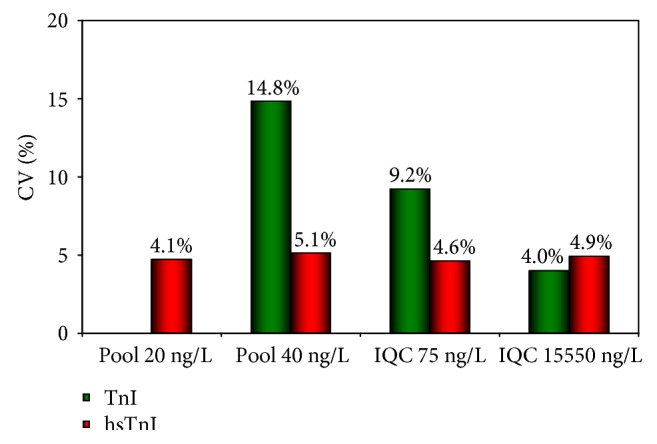
Total imprecision (CV%) for the TnI and hsTnI assays at four levels of internal controls assayed for the duration of the study. The 20 ng/mL pool has not been assayed by TnI as it is below the LoQ for this assay. IQC = internal quality control.

**Figure 2:**
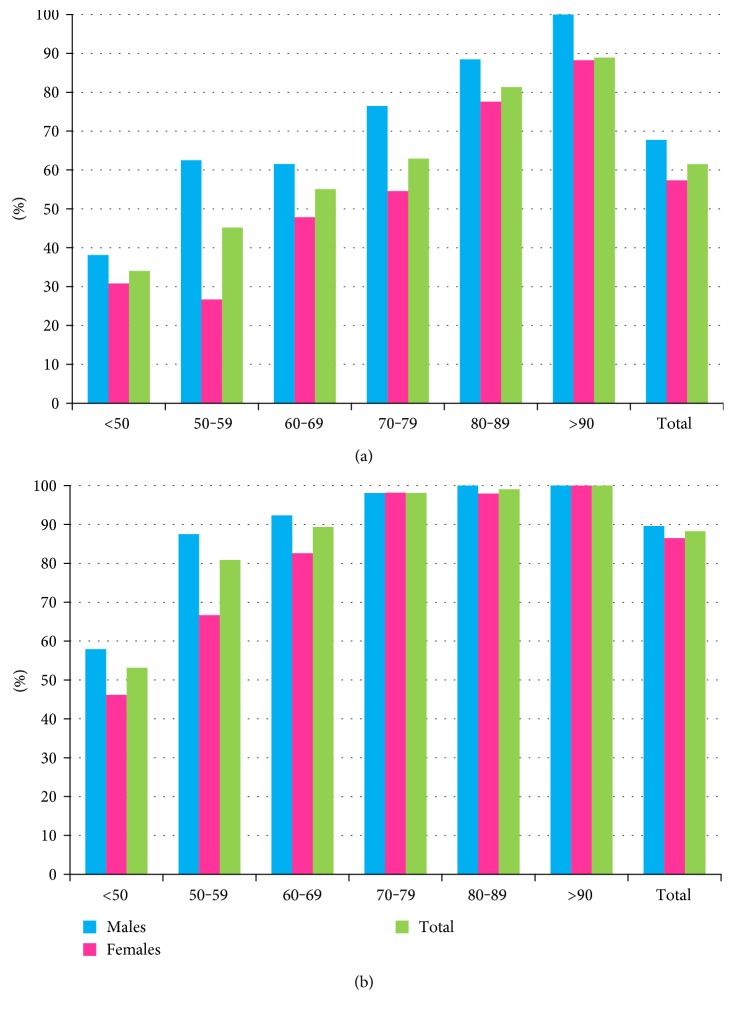
Rates of detection for cardiac troponin I by TnI (a) and hsTnI (b) assays by gender and age classes.

The total imprecision of TnI and hsTnI is reported in Figure 1. The hsTnI assay yielded a total CV <5% at all four ICQ levels, including the lowest one set at 20 ng/L, while TnI attained total CVs <10% only at the two highest levels. Troponin I was above the limit of detection (LoD) that represents the minimum amount of analyte likely to be reliably distinguished from the limit of blank, or background noise, in 68.6% of the 564 samples collected by TnI (Figure 2(a)) and in 89.9% by hsTnI (Figure 2(b)) (*p* < 0.001 by Fisher's exact test), and the detection rate increased with age. Of note, rates were higher in male patients for all age classes by TnI, whereas by hsTnI, a gender difference was observed only in patients aged less than 70 years. The adoption of different thresholds has led to a difference in the percentage of patients with a troponin value exceeding that threshold, those being 27.3% by TnI and 33.9% by hsTnI (*p* < 0.01). Not surprisingly, since the gender threshold for females, available only by the hsTnI assay, is set at 16 ng/L compared to that at 44 ng/L by TnI, 38 of 41 patients with initial positive values only by hs-cTnI were women.

**Figure 3:**
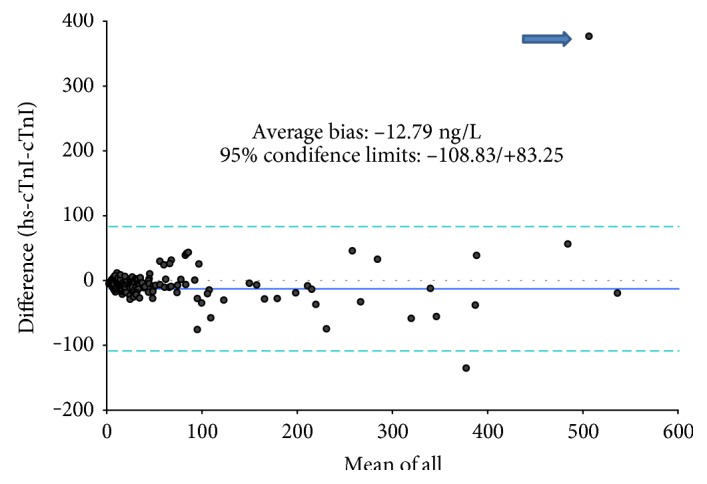
Bland–Altman difference plot (absolute values) between TnI and hsTnI in the range between 44 and 600 ng/L. One outlier (arrow) was observed.

The correlation between TnI and hsTnI was very good both by the Pearson and by Passing and Bablok methods (Pearson: *y* = 1.1095*x* − 113.52; *R*^2^ = 0.92; Passing and Bablok: *y* = −6.44 + 0.98*x*). The difference plot analysis according to Bland–Altman (Figure 3) has been carried out in the clinically more relevant range between 44 (LoQ of the TnI assay) and 500 ng/L and evidenced an average bias of −12.79 ng/L with (confidence interval: 21.93/−3.66) with 95% confidence limits between −108.83/+83.25), similar to the first comparative study between the two methods [7].

### 3.3. Clinical Results

A final clinical diagnosis of acute myocardial infarction (AMI) was established for 45 of the 426 patients (10.5%), with a significant difference (*p* < 0.05) between females (9.2%) and males (11.6%); these data are in lower end of the range (9.2–23.2%) reported by other studies [7–14]. The number and rates of different diagnoses are reported in Table 3; the most frequent ones being nonspecific chest pain (92 cases) and neurological diseases (74, mostly syncope). The high age of the study population (61.7% of patients being more than 65 years old) may help explain those findings. The compliance with the study protocol was not high: from 312 patients, or 72.3%, a single blood sample was obtained as those cases were either admitted immediately to clinical wards with a definite diagnosis (Table 3) or ruled out. Of the remaining 114 patients, 83 (72.8%) were tested for troponin after 3 (or 3 and 6) hours and 31 were assayed only after 6 hours.

Troponin levels above the adopted thresholds were found in 81.6% of AMI cases by TnI and in 83.7% by hsTnI (p = ns). This finding was quite frequent also in other disease groups, and especially among patients diagnosed with an acute heart failure (Table 3; 54.5% by TnI and 77.3% by hsTnI) thus confirming that an initial “positive” result for cardiac troponin has a quite low positive predictive value (PPV) for AMI. In our experience, the initial finding of a troponin I value above the thresholds had indeed a PPV of 39.0% by TnI and 28.7% by hsTnI (Table 4).

Serial testing, as suggested by the guidelines and confirmed in clinical practice [2, 3, 5–8], yields more accurate results as it guarantees that an ongoing process of myocardial necrosis is taking place; this, coupled with symptoms and signs related to or compatible with coronary artery disease, actually defines AMI [2]. According to the study protocol, the sensitivity, specificity, NPV, and PPV have been assessed at T3 and T6 for a TnI and hsTnI percent variation ≥50% (Table 4). The highest sensitivity (90.9%) was attained at T3 by both assays, with a NPV of 95.0% for TnI and 95.8% for hsTnI, while the highest specificity, and as a consequence PPV (100% for both), were reached by hsTnI at T6. Overall, the most efficient approach was guaranteed by hsTnI at both time intervals.

We observed nine cases overall for whom the shift in cardiac troponin values by one, or both assays did not correspond with the clinical diagnosis posed in the ED (Table 5). After admission to the clinical wards, mainly to Cardiology, the clinical judgment was revised and in 7 out of 9 AMI (6 NSTEMI, 1 STEMI) was diagnosed, with only two cases being still considered as “false positives.”

**Figure 4:**
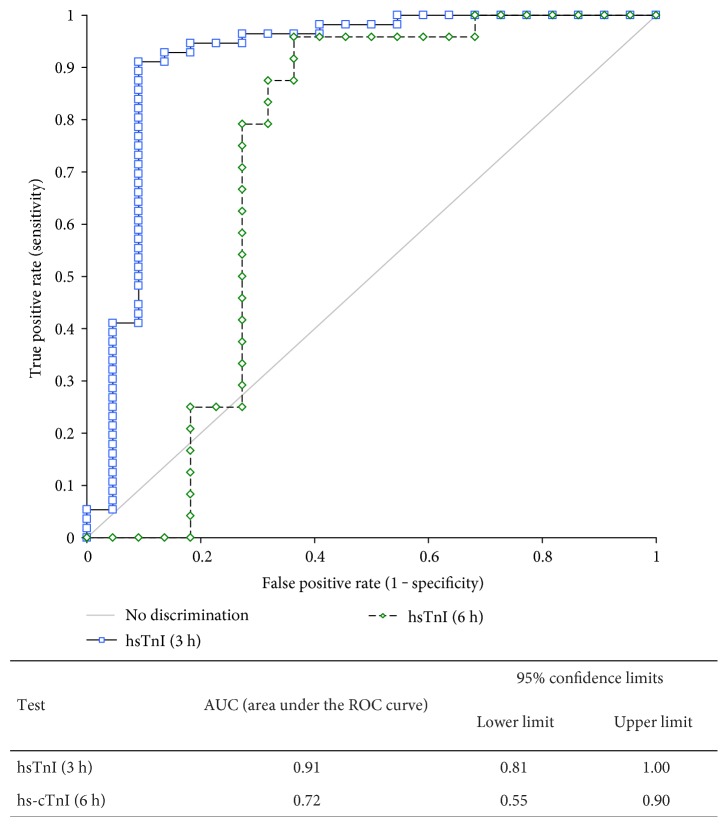
Receiver operating characteristics (ROC) curves for hsTnI according to an increase at 3 and 6 hours compared to baseline.

The ROC curve analysis demonstrated a higher accuracy of hsTnI by the 3-hour algorithm, yielding an AUC of 0.91, quite comparable to the 0.92 recently described by Collinson et al. [15], compared to the 0.72 by the 6-hour algorithm (Figure 4). The difference did not reach a statistical significance (*p* = 0.08) due to the low number of cases diagnosed with AMI.

### 3.4. Clinical Cases

**Figure 5:**
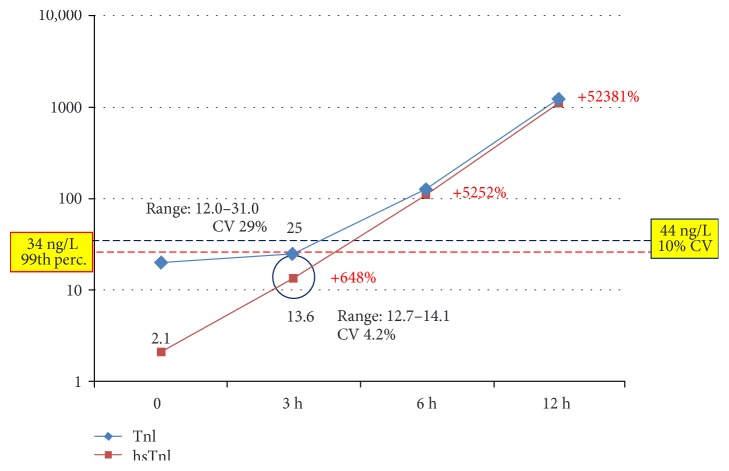
Clinical case 1. Increase of cardiac troponin I by the TnI and hsTnI assay in a male patient eventually diagnosed with NSTEMI. The imprecision for the results obtained at T3 was established by repeating each assay four times. Percentages in red represent the increase by hsTnI compared to baseline. CV = coefficient of variation. Red line: threshold for hsTnI; blue line: threshold for TnI.

**Figure 6:**
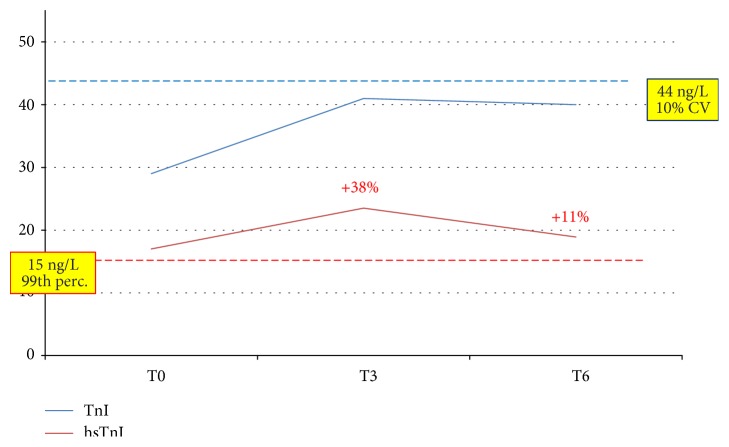
These patients (female, 82 years, acute hearth failure) would have been effectively ruled out for an acute coronary syndrome by the hsTnI assay after 3 hours, according to the ESC guidelines [2], but not by TnI. Percentages in red represent the increase compared to baseline. Red line: threshold for hsTnI; blue line: threshold for TnI.

**Figure 7:**
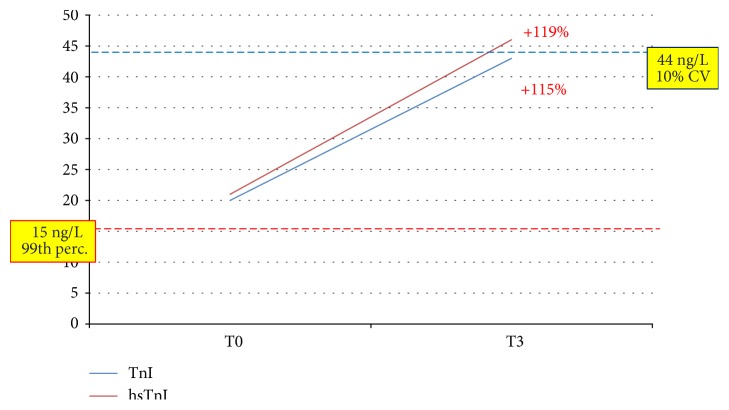
Clinical case 3. This 91-year-old female patient was one of the two “false positives” by hsTnI. The increase of TnI after 3 hours (bold red) was significant by both assays, but values above the diagnostic threshold (gender-specific 99th percentile) could be recorded only by hsTnI. Red line: threshold for hsTnI; blue line: threshold for TnI.

To highlight better the clinical implications of testing by hsTnI versus TnI, we describe here three clinical cases. The first one (Figure 5) was an 88-year-old male patient with a clinical history of coronary disease: troponin was detectable at baseline by the hsTnI assay (2 ng/L) and undetectable by TnI. At T3, both assays yielded a measurable result below the positivity threshold (28.3 ng/L by TnI and 13.6 ng/L by hsTnI), with an almost seven-fold increase by the latter. At T6, both assays indicated an ongoing cardiac necrosis by showing clearly positive values and a very significant increase compared to baseline; eventually, a diagnosis of NSTEMI was posed after a 15-hour stay in the ED. The adoption of an algorithm based on the kinetics of hsTnI would have substantially reduced the length of stay in the ED, and the same would have happened for the patient whose data are depicted in Figure 6. An 82-year-old female with cerebrovascular problems and suspected ACS, she had initially a normal result by TnI and a value above the gender threshold for hTnI; the latter did not show a significant increase after 3 hours (+38%) neither after 6 hours (+11% from baseline). According to the suggested protocol, being this patient at low risk, she could have been discharged after 3 hours. The third case (Figure 7) is one of the two “false positives” according to the hsTnI kinetics: a 92-year-old female, diagnosed with acute respiratory failure, showed a significant increase of TnI after 3 hours by both assays (+ 119% and +115%, respectively), but values above the diagnostic threshold (gender-specific 99th percentile) could be recorded only by hsTnI. Since the clinical decisions were based upon the current standard of care, AMI was ruled out though it would have been diagnosed according to the 3rd universal definition of AMI and the recent ESC guidelines [2, 3].

### 3.5. New ESC 1-Hour Algorithm

**Figure 8:**
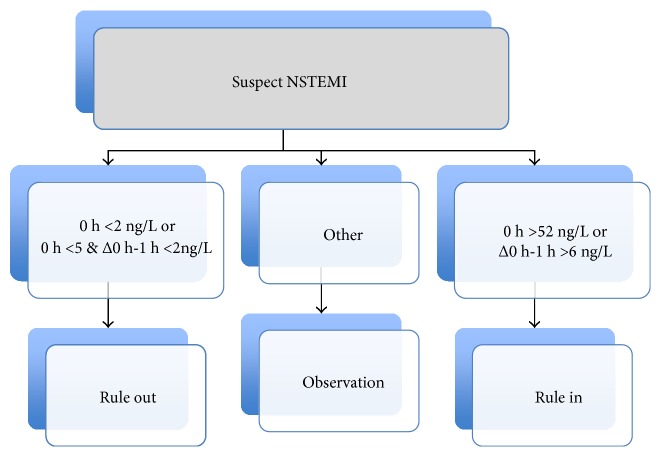
Accelerated (0 and 1 hour) testing algorithm from the ESC 2015 guidelines. Values refer to the Architect hsTnI assay results.

The new ESC guidelines for NSTEMI suggest that, whenever a high sensitivity assay is available, the time span between the baseline test for troponin and the following one may be reduced to 1 hour and the difference in cardiac troponin concentration over time shall be measured in absolute values (ng/L) and not in percentage (Figure 8) [3]. Indeed some recent publications have documented this approach as being clinically accurate, especially for ruling out [12–14, 16]. Even if in our experience the second draw was obtained after 3 hours, we tried to apply this “accelerated” algorithm by using only the results obtained by the hsTnI assay at baseline. The results (Table 6) indicate an absolute sensitivity and NPV (both 100%) for AMI. Conversely, the PPV was low (13.8%) as the majority of patients not suffering from AMI (75.3%) would have been included in the “Observation” group.

### 3.6. Follow-Up

**Figure 9:**
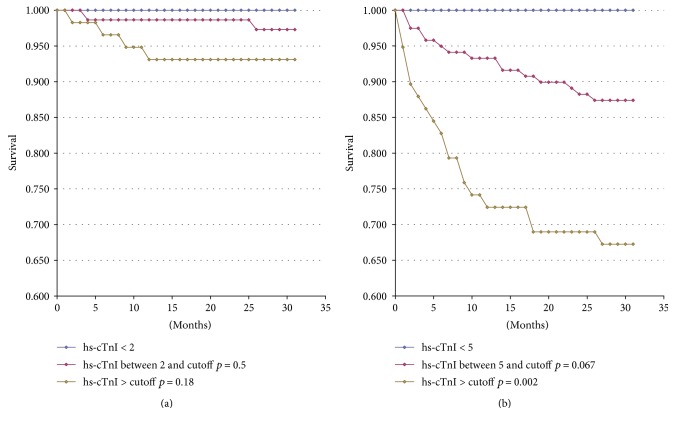
Mortality due to acute coronary syndrome (a) and all-cause mortality (b) after 18 months from enrollment in the study according to baseline hsTnI values on the 202 patients enrolled at the Civil Hospital in Sondrio.

Cardiac troponin represents also a prognostic marker for morbidity and mortality, both for cardiac ischemic disease and for cardiovascular diseases as a whole and for other causes. This is not surprising, since higher circulating levels of troponin indicate remodeling and/or chronic damage of the cardiac muscle [17]. For the 202 patients enrolled at one of the two sites (Sondrio), we were able to access the official regional mortality registry to check out the survival after 18 months from the study period (Figure 9) considering cardiovascular mortality and all-cause mortality in relationship with the baseline hsTnI values. Those did not reach the statistical significance for cardiovascular mortality either for values between 5 ng/mL and the gender threshold or for those above the latter (Figure 9(a)) due to the low frequency of events over the follow-up period. On the other hand, there was a significant relationship (*p* = 0.02) between hsTnI values exceeding the gender-specific 99th percentile and all-cause mortality (Figure 9(b)).

## 4. Conclusions

In Western countries, including Italy, access to the EDs has remarkably increased due to insufficient resources for extra-hospital treatment of acute patients and to the ageing of the resident population that leads to a higher number of elderly people presenting to the ED with acute exacerbations of chronic diseases. It has been estimated that 10–15% of ED patients present with chest pain or other signs suggestive of myocardial ischemia, but a final diagnosis of ACS can only be made in 15–25% of them, which overall represents the 2–5% of all incomers [1, 16, 18]. A rapid rule out of AMI will then be beneficial both for patients and for ED personnel and may also result in health cost savings [1]. The results we have described here confirm that ruling out by the hsTnI assay may be safe and effective. On this purpose, the adoption of the 99th percentile of a normal population has been deemed not to be safe enough: according to Pickering et al. [11], the sensitivity of the 99th percentile to rule out AMI was only 93.2%, and several studies have addressed this issue by choosing a much lower value still measurable by the hsTnI assay. In a classic prospective study, Shah et al. [9] have adopted a ruling out threshold on the baseline sample at 5 ng/L that guaranteed a NPV of 99.4% and allowed to dismiss safely almost two thirds of suspected cases. Similar results have been described by Neumann et al. [13] with a threshold at 6 ng/mL; moreover, at this concentration, TnI had also a greater predictive value for 1 year mortality that was registered in 1% of cases compared to 3.7% for patients with troponin levels below the 99th percentile. A more extreme approach indicates that an undetectable troponin level at baseline (i.e., <2 ng/L by the Architect hsTnI assay) would guarantee an even greater NPV for AMI. This is indicated in the aforementioned ESC guidelines [3] and has been proved effective in subsequent studies [12, 14].

On the other hand, since troponin testing has gained a central role for the diagnosis of AMI, ruling in shall be also taken care of with a keen eye on establishing the most effective algorithms. On this, a quite relevant issue is represented by gender differences: it has been long known that cardiac troponin levels are lower in women, but until the high sensitive assays for cardiac troponin have been made available, those differences could not be implemented in clinical practice, since the limit of quantitation of commercial assays was higher than the 99th percentile in women. The high sensitive assay we have described here does provide robust gender differences, both values being much higher that LoQ, and has been already demonstrated to allow a much better diagnosis of AMI in women: Shah et al. reported a 100% increase in true diagnosis in the female gender by shifting from TnI to hsTnI and adopting gender specific thresholds [19], and even our limited experience allowed us to find et least one case that would have been better defined by hsTnI according to the current clinical standards [2, 3].

**Figure 10:**
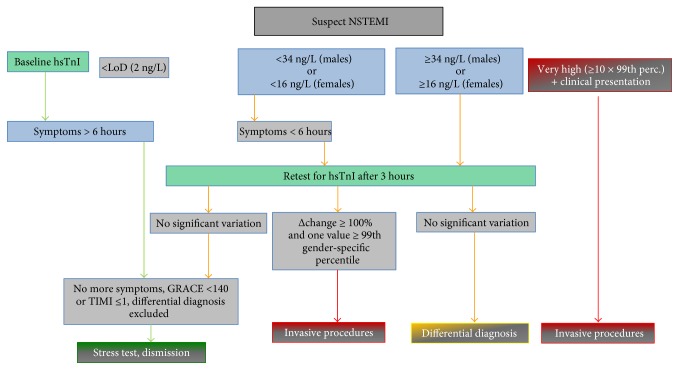
Diagnostic algorithm for patients with suspected NSTEMI adopted at both study sites after the experimental phase with hsTnI. The algorithm has been derived from the ESC 2015 Guidelines [3] and takes in account also the time from the onset of symptoms.

Taking all this in account, and also considering the results we have obtained, after a joint revision of the study results and outcomes with the Medical Officers and the Cardiology and ED Ward Directors, we have decided the shift from TnI to hsTnI by the protocol described on Figure 10. This is mostly taken from the ESC guidelines [3], with three differences: the adoption of the limit of detection on the rule-out side, establishing as “very high” initial values those at least tenfold the 99th percentile, as compared to 5-fold in the guidelines, and the definition of a 100% variation of troponin I concentration on the second sample as a decisional cutoff. For the last one, when we assessed the relative accuracy of hsTnI, the AUC obtained at 3 hours by a 50% (74.7%) and a 100% increase (80.2%) were not statistically different but, being the latter higher, we decided to adopt a 100% raise/fall threshold and possibly to change it later on after having reviewed the routine results obtained over at least one year from the implementation.

Though this study represents one of the very few available evidences on the “real life” transition from a contemporary sensitive to a high sensitive troponin assay, we need to underline some limitations. First of all, the number of AMI cases that have been eventually diagnosed was quite low and furthermore also the compliance to the study protocol was not high, since on many patients testing after 3 hours from baseline has not been performed. Both factors may have reduced the positive impact of hsTnI both on ruling out and in ruling in. Also, having limited the follow-up to 202 out of 426 patients may have reduced the strength of the association between initial TnI levels and fatality rates.

The approach we described here was well accepted by all departments involved and allowed, in quite a short time, a smooth transition to the high sensitivity assay for cardiac troponin I. Furthermore, it fostered the relationship between laboratory services and clinical departments and shall represent the pillar for future evaluations aimed to be a better, evidence-based, and possibly more sustainable health care.

## Figures and Tables

**Table 1:** Main features of the assays for cardiac troponin I (TnI) employed for the study. n.a. = not available.

	Contemporary TnI	High sensitive TnI
Limit of blank (LoB)	n.a.	0.7–1.3 ng/L
Limit of detection (LoD)	<10 ng/L	1.1–1.9 ng/L
Limit of quantitation (LoQ)	<100 ng/L (adopted: 44 ng/L)	4.0–10 ng/L
Gender-specific 99th percentile	n.a.	Females: 15.6 ng/mL
		Males: 34.2 ng/L

**Table 2:** Main demographic characteristics of the study population. SD = standard deviation.

Gender	*N*	%	Mean age (years)	SD	Median age (years)	% >65 years old	% admitted to hospital wards
Female	185	43.4%	70.9	17.2	76	70.8%	32.7%
Male	241	56.6%	67.3	16.7	69	54.8%	39.2%
Total	426	100.0%	68.8	17.0	73	61.7%	36.4%

**Table 3:** Final diagnosis for all patients and percentages of patients with a single blood draw and a baseline (T0) troponin I value above the adopted threshold by TnI and hsTnI. ACS = acute coronary syndrome; HF = heart failure; GI = gastrointestinal; NSCP = nonspecific chest pain.

Diagnosis	*N*	Single blood draw	cTnI above threshold at T0	hs-cTnI above threshold at T0
ACS	45	11.1%	81.6%	83.7%
Severe arrhythmia	45	75.6%	24.4%	31.1%
Acute HF	22	54.5%	54.5%	77.3%
Lung disease	46	87.0%	21.7%	37.0%
Neurological disease	74	86.5%	6.8%	10.8%
GI disease	46	82.6%	10.9%	15.2%
NSCP	92	58.7%	4.3%	7.6%
Other	56	83.9%	5.4%	21.4%
Total	426	69.0%	19.2%	27.0%

**Table 4:** Sensitivity, specificity, and positive and negative predictive values (PPV, NPV) according to TnI and hsTnI results at T0 and percent delta changes from baseline (Δc) after 3 and 6 hours from baseline (T3 and T6).

	Sensitivity	Specificity	PPV	NPV
T0				
TnI	71.0%	86.9%	39.0%	96.2%
hsTnI	73.3%	78.5%	28.7%	96.1%
T3				
ΔcTnI	90.9%	67.9%	52.6%	95.0%
Δc hsTnI	90.9%	82.1%	66.7%	95.8%
T6				
ΔcTnI	83.3%	85.7%	78.9%	88.9%
Δc hsTnI	83.3%	100%	100%	90.3%

**Table 5:** Details on nine cases with initial diagnosis in the Emergency Department discrepant with cardiac troponin I results. The final diagnosis (last column on the right) was consistent with an acute coronary syndrome in seven of them. The last case is described in more detail on Figure 7. ED = emergency department; n.a. = not available; APT = atrial paroxysmal tachycardia; cTnI and hs-cTnI: assay results by contemporary and high sensitive assays for cardiac troponin I, expressed in ng/L; STEMI = myocardial infarction with ST elevation; NSTEMI = myocardial infarction without ST elevation; APE = acute pulmonary edema.

Sex	Age	Initial diagnosis (ED)	TnI T0	hsTnI T0	TnI T3	hsTnI T3	TnI T6	hsTnI T6	TnI increase from T0	Final diagnosis
F	91	Acute pulmonary edema	371	409	n.a.	n.a.	1366	1523	172%	NSTEMI (exitus)
M	81	Acute pulmonary edema	213	117	n.a.	n.a.	8760	11,621	9699%	APE + STEMI
M	56	Acute pericarditis	222	208	842	991	n.a.	n.a.	376%	NSTEMI + multiple pathologies
M	64	Acute pulmonary edema	42	32	123	111	121	112	147%	APE + NSTEMI
F	75	Anaphylactic shock	39	30	134	104	n.a.	n.a.	247%	NSTEMI (referred to other hospitals)
F	88	APT	61	63	236	n.a.	346	334	283%	NSTEMI + APT
F	80	Acute pulmonary edema	51	45	n.a.	n.a.	137	123	73%	APE + NSTEMI
M	89	Lipothymia	40	14	62	35	n.a.	n.a.	93%	No ACS—pacemaker implanted
F	91	Acute respiratory failure	20	21	43	46	n.a.	n.a.	116%	Acute respiratory failure

**Table 6:** Presumptive diagnosis based on the results for hsTnI on the first draw (baseline) according to the ESC guidelines [2]. AMI = acute myocardial infarction. Numbers in brackets represent percentages of total cases indicated in the last column.

	Rule in (hsTnI >52 ng/L)	Observation	Rule out (hsTnI <2 ng/L)	Total cases
ACS	28 (62.2%)	17 (37.8%)	0	45
No ACS	40 (10.5%)	287 (75.3%)	54 (14.2%)	381
